# Leprosy, the Great Imitator of Rheumatic Diseases: A Case Study

**DOI:** 10.7759/cureus.39527

**Published:** 2023-05-26

**Authors:** Haidy Youssef, Tandis Mahani, Mehrnaz Hojjati

**Affiliations:** 1 Rheumatology and Internal Medicine, Riverside Medical Clinic, Temescal Valley, USA; 2 Internal Medicine, University of California, Riverside, USA; 3 Rheumatology and Internal Medicine, Loma Linda University School of Medicine, Loma Linda, USA

**Keywords:** mctd, cutaneous vasculitis, hansen’s disease, vasculo-necrotic reaction, systemic lupus erythema, rheumatoid arthriitis, lepromatous leprosy

## Abstract

A 68-year-old Hispanic man was referred to our center for cutaneous vasculitis of the lower extremities, diagnosed via skin biopsy. He had a 10-year history of erythematous plaques complicated by persistent, non-healing ulcers previously treated with prednisone and hydroxychloroquine. Laboratory testing was significant for positive U1-ribonucleoprotein antibody, antinuclear antibody human epithelial-2, and an elevated erythrocyte sedimentation rate. A repeat skin biopsy revealed nonspecific ulcerations. The patient was diagnosed with a mixed connective tissue disease with features of scleroderma. Mycophenolate was initiated, and prednisone was tapered. After two years of relapsing ulcerations on his lower extremities, a third skin punch biopsy showed dermal granulomas with numerous acid-fast organisms, and a polymerase chain reaction identified *Mycobacterium lepromatosis*, indicating polar lepromatous leprosy with an erythema nodosum leprosum reaction. After three months of minocycline and rifampin therapy, his lower extremity ulcerations and erythema resolved. Our case highlights the variable and elusive nature of this disease, which can mimic many systemic rheumatologic conditions.

## Introduction

Leprosy, also known as Hansen's disease, is an infectious disease caused by *Mycobacterium leprae* and *Mycobacterium (M.) lepromatosis* that primarily affects the skin and peripheral nerves.

Clinical manifestations of leprosy are highly variable, and given its multisystemic nature, it can mimic many systemic rheumatologic diseases. A hallmark of leprosy is the presence of hypopigmented or erythematous macules or papules, which are often associated with neuropathy due to the infecting organisms’ high tropism for peripheral nerves [1]. Nearly half (30-50%) of people with leprosy experience immunologic reactions before, during, or after treatment [2]. Over the past two decades, leprosy has been repeatedly misdiagnosed as vasculitis, lupus, rheumatoid arthritis, sarcoidosis, and other autoimmune conditions [3-9]. Here, we present the case of a 68-year-old Hispanic male referred to us for recurrent cutaneous vasculitis of the lower extremities.

## Case presentation

**Figure 1:**
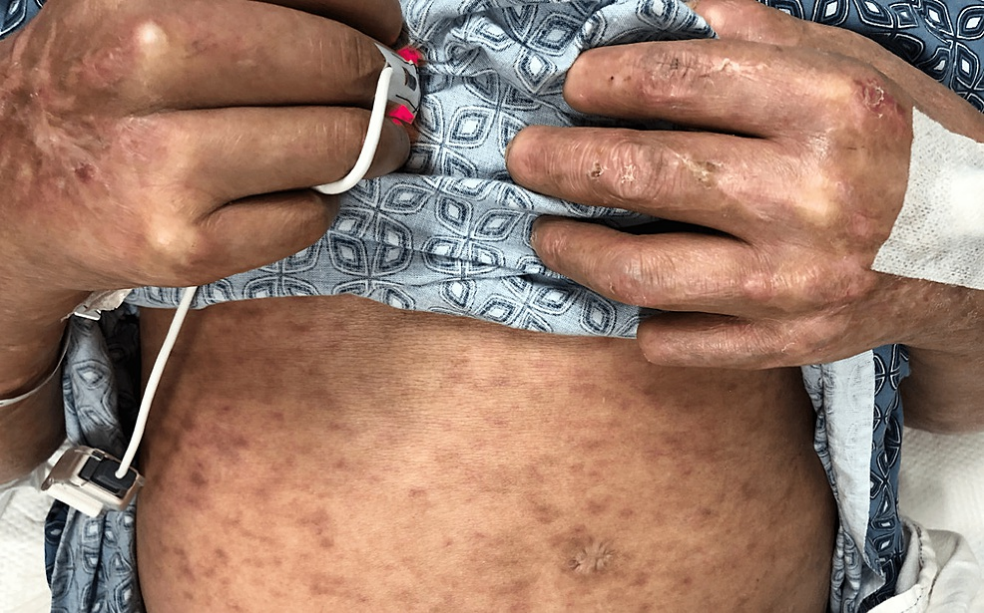
Hypo- and hyperpigmented plaques

**Figure 2:**
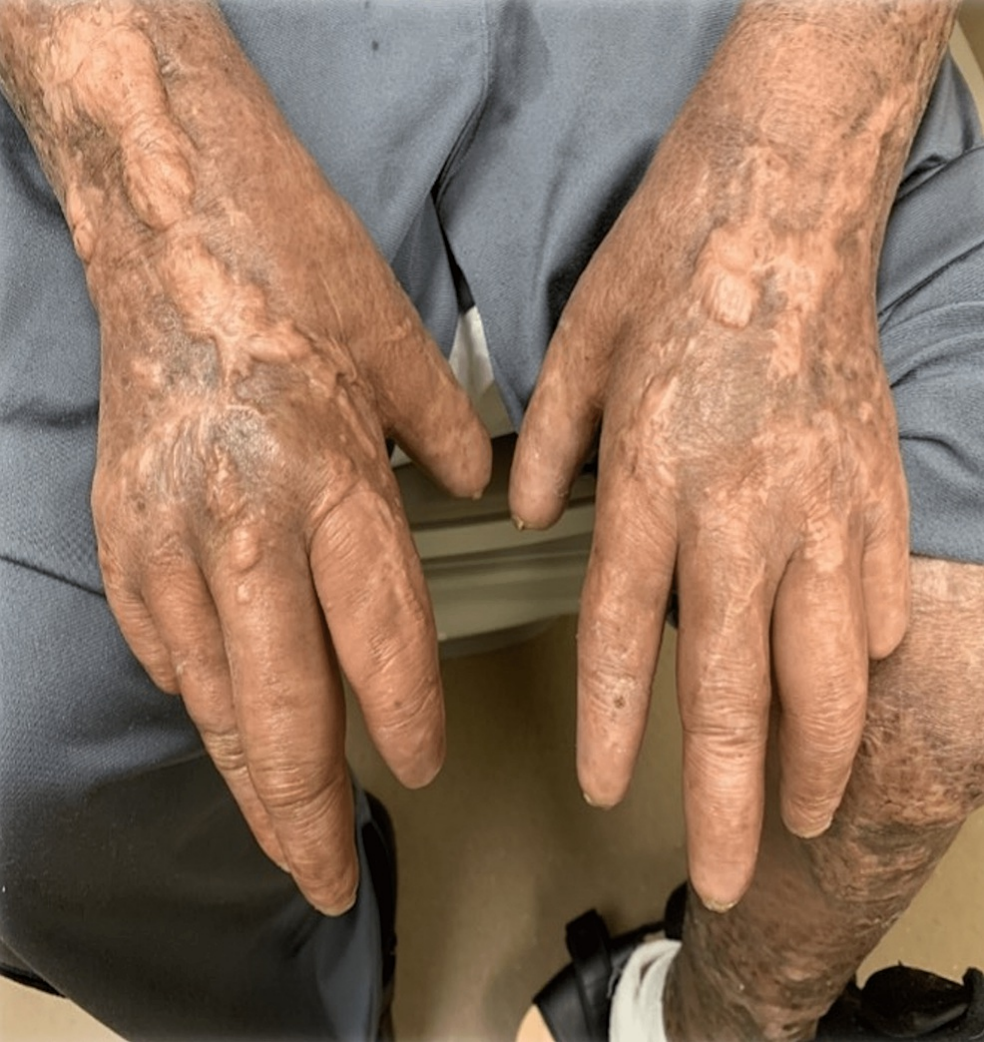
Shortening of fingertips and sclerotic skin changes

**Figure 3:**
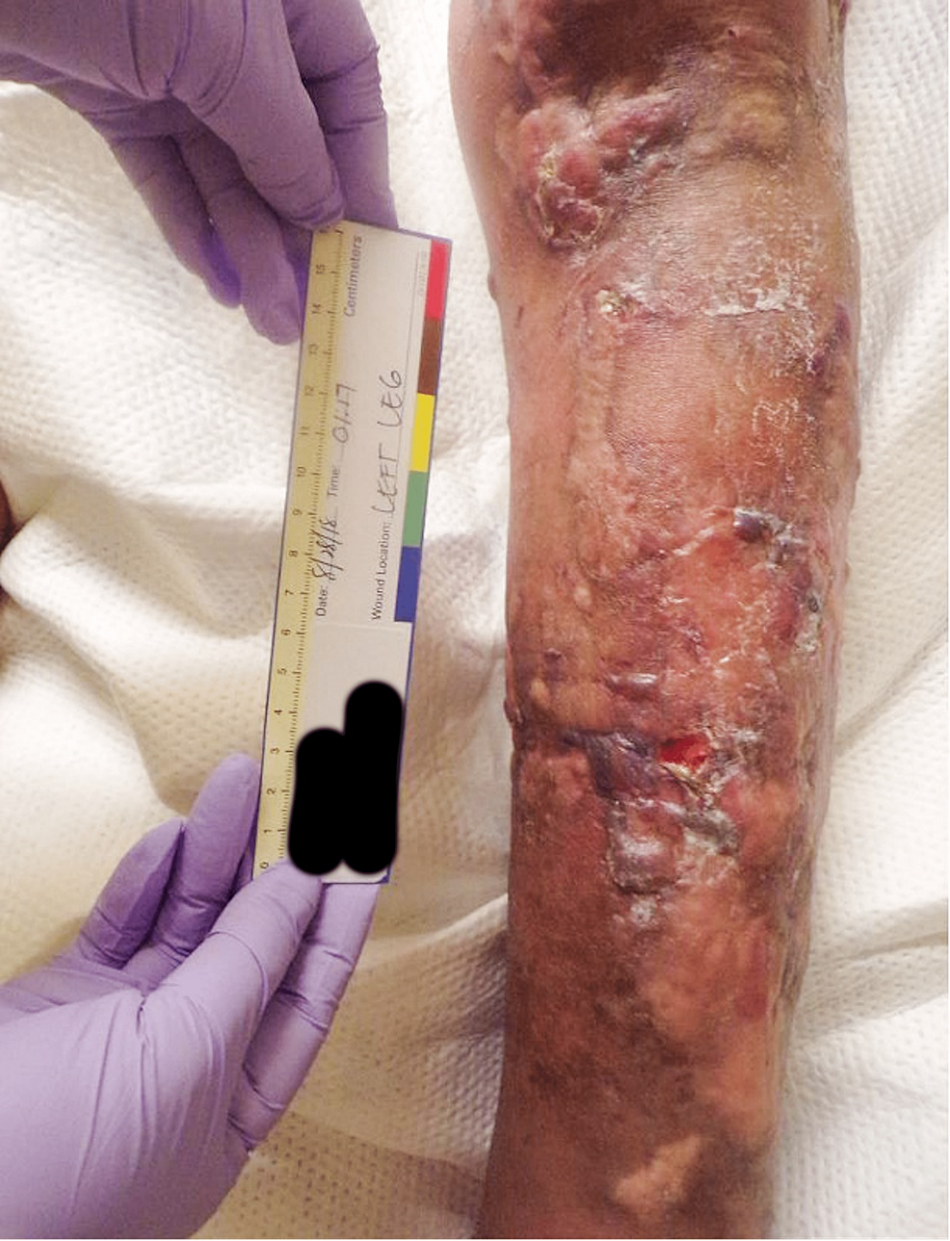
Ulcers and hypo- and hyperpigmented plaques with sclerotic skin changes

A 68-year-old Hispanic man was referred to our center for cutaneous vasculitis of the lower extremities, diagnosed via skin biopsy. He reported a 10-year history of erythematous plaques, mainly on his lower legs, complicated by persistent, non-healing ulcers. The treatment history included prednisone and hydroxychloroquine. He also reported multiple cuts and burns on his hands due to a lack of sensation for pain and temperature, prominent paresthesia across all extremities, and diffuse joint pain. His exam revealed coarsened facial features, saddle nose deformity, ectropion with scleral injection, and loss of eyebrows. His hands revealed acro-osteolysis with shortened fingertips, skin tightening, and tenosynovitis of all digits. The skin exam was remarkable for non-healing ulcerations with hypo- and hyperpigmented indurated plaques and sclerotic skin changes on all extremities, particularly the legs (see Figure 1,2,3). He had a diminished pinprick sensation noted in both the upper and lower extremities.

Laboratory testing showed elevated levels of U1-ribonucleoprotein antibodies at 50.8 U (normal level < 20 U), rheumatoid factor at 42 IU/mL (normal level < 15 IU/mL), and anticardiolipin immunoglobulin M at 28 MPL U/mL (normal level < 12.5 MPL U/mL), as well as a positive antinuclear antibody by hep-2 assay at 1/160 speckled pattern and an elevated erythrocyte sedimentation rate of 65 mm/hr (normal level < 20 mm/hr). Other immunology labs, as well as the hepatitis serology, HIV, QuantiFERON, venereal diseases, and coccidioidomycosis assays, were all unremarkable. A repeat skin biopsy revealed nonspecific ulcerations. According to clinical and lab findings, the patient was diagnosed with mixed connective tissue disease (scleroderma variant).

**Figure 4:**
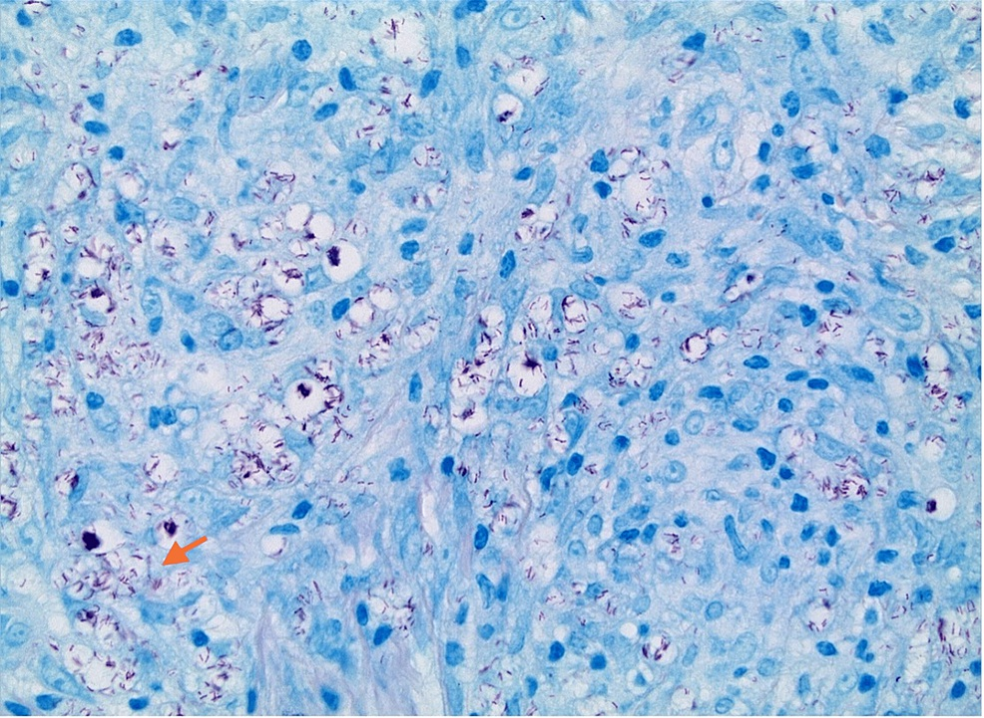
Skin biopsy with Fite stain acid-fast 50x magnification, showing numerous organisms within granulomas

He was started on prednisone (20 mg/day) and hydroxychloroquine (Plaquenil, 400 mg/day). Later, mycophenolate (2000 mg/day) was added, and the prednisone was tapered. Throughout two years of follow-up, he continued to have relapsing, non-healing ulcerations of his lower extremities, along with dysesthesia in his extremities. Due to refractory manifestations while on immunosuppressive therapy, a repeat skin punch biopsy was taken from his right lower leg near the edge of a blister. The culture came back significant for dermal non-caseating granulomas with numerous acid-fast organisms (see Figure 4). Foamy granulomas and perivascular involvement were detected, suggesting leprosy. Immunofluorescence staining of the ulcer tissue was negative for immunoglobulin A (IgA), IgG, IgM, and C3. A polymerase chain reaction analysis at the National Hansen's Disease (Leprosy) Clinical Center in Baton Rouge, Los Angeles, confirmed *M. lepromatosis*. The patient was diagnosed with polar lepromatous leprosy with erythema nodosum leprosum reaction. Minocycline and rifampin were initiated. Both mycophenolate and hydroxychloroquine were discontinued, and low-dose prednisone (5 mg/day) was continued for immunologic symptoms. Three months into therapy, his lower extremity ulcerations and erythema had resolved. At his six-month follow-up, his arthritis and profound dysesthesia had significantly improved.

## Discussion

Leprosy, or Hansen's disease, is a chronic infectious granulomatous disease caused by the acid-fast bacilli *M. leprae and M. lepromatosis*. According to the Registry of the National Hansen's Disease Programs, 205 new cases of leprosy were detected in the United States in 2010, indicating the disease is not as rare in developed countries as previously thought [10]. As leprosy tends to be chronic with constitutional symptoms and multisystemic manifestations, it can mimic systemic rheumatologic conditions. According to a case series by Salvi et al., 1-5% of patients with leprosy develop arthritis of the small joints of the hands and feet, similar to those with rheumatoid arthritis [9], but erosive arthritis is not found on radiological examination. Although several autoantibodies (e.g., rheumatoid factor and antinuclear antibodies) and ribonucleoprotein antibodies may be positive in leprosy [11], anti-cyclic citrullinated peptide( anti-CCP ab), which has a higher specificity and diagnostic value in rheumatoid arthritis, has not been reported in leprosy patients with arthritis.

Patients with leprosy can present with two types of immune-mediated reactions [12].

The type I reaction is a class IV hypersensitivity reaction with heightened cell-mediated immunity and is seen in borderline forms of leprosy. When occurring prior to therapy, the patient worsens and develops features of lepromatous leprosy: fever, edema, inflammatory skin lesions, and severe painful neuritis manifesting as tender peripheral nerves, foot drop, claw hand, and facial palsy [9]. Type II leprosy and the Lucio phenomenon are vasculo-necrotic reactions that mimic primary vasculitic conditions. The Lucio phenomenon is a type III hypersensitivity presented exclusively in (typically untreated) diffuse lepromatous leprosy and is characterized by immune complexes, necrotizing vasculitis on superficial and medium-sized vessels, diffuse infiltration of the skin, dermal necrosis, and sometimes systemic symptoms [13]. Histologically, it appears as polymorphic nuclear vasculitis or panniculitis [9].

Given the symptoms, patients usually present to dermatology settings first and less commonly to outpatient rheumatology settings, as in our case. Along with the previous case reports listed in Table 1, the case described here bears significant resemblance to multiple rheumatologic conditions. Leprosy thus should be considered a differential diagnosis in patients presenting with rheumatic and cutaneous manifestations, especially if they have other risk factors (e.g., travel to endemic areas like India, Brazil, Indonesia, Bangladesh, Nigeria, and Mexico; close contacts with people with leprosy [14], immunosuppression [15], HIV [16], or chemotherapy [17]).

**Table 1:** Summary of case reports in the literature initially diagnosed as rheumatologic conditions before a final diagnosis of leprosy SpA: spondylarthritis; PsA: psoriatic arthritis; NSAID: non-steroidal anti-inflammatory drugs; RA: rheumatoid arthritis; MCP: metacarpophalangeal; PIP: proximal interphalangeal; SSc: systemic sclerosis; ANA: antinuclear antibody; SLE: systemic lupus erythematosus; HCQ: hydroxychloroquine; MTX: methotrexate

Author	Sex, age at 1^st^symptom	Initial symptoms	Initial diagnoses	Initial treatment	Time to correct diagnosis of lepromatous leprosy	Leprosy treatment
Ramos et al. [3]	80 Female	Painful ulcers on extremities, erythematous plaques, fatigue	Vasculitis	Prednisolone	8 months	Clofazimine, dapsone, rifampicin
Rath et al. [4]	40 Male	Joint pain, red plaques over back and left knee	Spondylarthritis, psoriatic arthritis	Steroids, sulfasalazine, NSAIDs	2.5 years	Multidrug therapy, steroid
Simeoni et al. [5]	20 Male	Erythematous skin macules, fever, arthritis, episcleritis	Sarcoidosis; adult-onset Still’s disease, dermal reaction to hydroxychloroquine	High-dose steroids, methotrexate, hydroxychloroquine	2 years	Rifampicin, clofazimine, dapsone plus prednisolone
Sampaio et al. [6]	86 Female	Polyarthritis (metacarpophalangeal, proximal interphalangeal, wrists, elbows)	Rheumatoid arthritis	Prednisolone, naproxen	5 years	Rifampin, dapsone, clofazimine
Lee et al. [7]	82 Female	Erythematous plaques with hemorrhagic necrosis, hypoesthesia, and Raynaud’s phenomenon	Beurger’s disease, systemic sclerosis	Symptomatic	6 years	Multidrug leprosy treatment
Horta-Baas et al. [8]	50 Female	Skin rash on face, extremities, and back; leukopenia, elevated antinuclear antibody at 1:80 speckled pattern; arthralgia; discoid rash; neuropathy; photosensitivity	Cutaneous lymphoma, sarcoidosis, lupus tumidus	Symptomatic prednisone, hydroxychloroquine NSAIDs	4 months	Dapsone, rifampin, clofazimine, prednisone
Salvi et al. [9]	52 Female	Symmetrical polyarthritis	Rheumatoid arthritis	Leflunomide 10 mg, Prednisolone 5 mg	6 months	Anti-leprosy drugs, low dose prednisone

## Conclusions

Patients with leprosy can present with predominant rheumatic manifestations, as shown in our case, initially diagnosed as cutaneous vasculitis and later as mixed connective tissue disease with refractory manifestations to glucocorticoids and immunosuppressive therapy. Multibacillary leprosy was finally confirmed after the third skin biopsy. Administration of a multidrug therapy led to a favorable response in the patient. Our case underscores the importance of considering infectious mimics of rheumatologic diseases, as early diagnosis increases the chance of full recovery and decreases morbidity.
